# Mantle upwelling at Afar triple junction shaped by overriding plate dynamics

**DOI:** 10.1038/s41561-025-01717-0

**Published:** 2025-06-25

**Authors:** Emma J. Watts, Rhiannon Rees, Philip Jonathan, Derek Keir, Rex N. Taylor, Melanie Siegburg, Emma L. Chambers, Carolina Pagli, Matthew J. Cooper, Agnes Michalik, J. Andrew Milton, Thea K. Hincks, Ermias F. Gebru, Atalay Ayele, Bekele Abebe, Thomas M. Gernon

**Affiliations:** 1 School of Ocean and Earth Science, University of Southampton, Southampton, UK; 2 Department of Mathematics and Statistics, Lancaster University, Lancaster, UK; 3 Dipartimento di Scienze della Terra, Università degli Studi di Firenze, Firenze, Italy; 4 GEOMAR Helmholtz Centre for Ocean Research Kiel, Kiel, Germany; 5 School of Cosmic Physics, Geophysics Section, Dublin Institute for Advanced Studies, Dublin, Ireland; 6 Dipartimento di Scienze della Terra, Università di Pisa, Pisa, Italy; 7 Department of Geosciences, University of Fribourg, Fribourg, Switzerland; 8 School of Earth Sciences, Addis Ababa University, Addis Ababa, Ethiopia; 9 Institute of Geophysics Space Science and Astronomy, Addis Ababa University, Addis Ababa, Ethiopia; 10 GFZ Helmholtz Centre for Geosciences, Potsdam, Germany; 11 Present Address: Geography Department, Swansea University, Swansea, UK

**Keywords:** Volcanology, Tectonics, Geodynamics, Geochemistry

## Abstract

Mantle upwellings drive large-scale surface volcanism and facilitate continental breakup and ocean basin formation. However, the spatial characteristics and internal composition of these upwellings alongside how they are modified by plate tectonics are poorly resolved. Afar, East Africa, is a classic triple junction comprising three rifts at various stages of evolution thought to be underlain by a mantle upwelling or plume, allowing examination of the controls on the mantle upwelling. Here we present geochemical data from >130 samples of ‘young’ volcanoes spanning the rifts defining the triple junction to show that the underlying mantle comprises a single, asymmetric upwelling. Using statistical modelling to integrate our data with existing geochemical and geophysical constraints, we suggest that Afar is fed by a spatially and chemically heterogeneous upwelling, which controls the composition and relative abundance of melt in all three rift arms. We identify repetitive signatures in mantle compositions in rift regions, whose variability is a longer wavelength in faster-extending rift arms. This suggests more rapid channelized mantle flow occurs where rifting rates are higher and the plate is thinner, aiding flow of the upwelling towards the faster-spreading Red Sea Rift. Our findings demonstrate how the evolution of mantle upwellings is influenced by the dynamics of overriding plates.

## Abstract (web summary)

The mantle upwelling beneath the Afar rift may be influenced by tectonic processes in the overriding lithospheric plates that shape the distribution of both the compositional heterogeneities and abundance of melt, according to a geochemical and statistical study of volcanic samples.

## Main

The role of mantle upwellings, sometimes interpreted as mantle plumes, in driving volcanism during continental breakup has long been debated (for example, refs. ^1–4^). Moreover, our understanding of rift–plume interactions remains incomplete because only a small fraction of Earth’s upwellings are situated under continents^5^, and there are a limited number of upwellings associated with ongoing continental rifting^6^. The Afar triple junction—where the Arabian, Nubian and Somalian tectonic plates intersect—is a ‘classic’ example of magma-assisted continental rifting. Here rifting occurred diachronously with the onset of the Gulf of Aden Rift (GoA) at ~35 million years ago (Ma) (ref. ^7^), the Red Sea Rift (RSR) at ~23 Ma (ref. ^8^) and the Main Ethiopian Rift (MER) at ~11 Ma (ref. ^9^). Both intraplate stresses tied to the slab pull effect of Neo-Tethys subduction^10^ and thermal weakening by a mantle upwelling are thought to have driven rifting^11^. The diachronous onset has led to each rift (GoA, RSR and MER) being in a different phase of maturity (ocean formation, proto-oceanic formation and mature continental rifting, respectively), and all three rifts are currently volcanically and tectonically active^12^, making it an ideal location to study the interactions between mantle upwelling and rifting and how these coevolve.

The driver of melt production in Afar is debated, with some models suggesting decompression melting with minimal plume involvement^13^, whereas others propose the upwelling of hot, deep mantle^14–17^ or, indeed, multiple upwellings^18,19^. While several discrete segments of the RSR have been studied in terms of magma petrogenesis (for example, refs. ^16,20^), a paucity of high-precision geochemical data has hampered evaluation of the spatial characteristics of upwelling across the broader region and rigorous testing of existing models of the links between tectonics and upwellings.

**Fig. 1:**
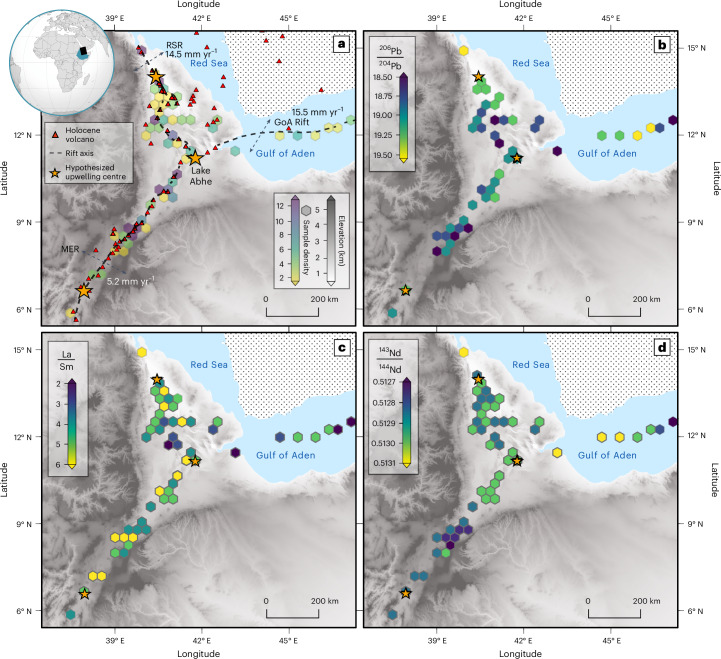
Variation in geochemical and geophysical properties around the Afar Triangle. **a**, The GoA, RSR and MER axes (dashed lines) and associated rifting rates indicated by arrows (from refs. ^42,47^). The three hypothesized^19,36,37^ upwelling locations (yellow stars) and Holocene volcanoes (red triangles) are shown. Hexmap colours show the density of samples within the hexagon’s area, with purple representing >12 and yellow showing 1–2. Location of maps shown on global inset (black rectangle). **b**, The ^206^Pb/^204^Pb variations across the study region (dark blue, low ^206^Pb/^204^Pb—minimal upwelling signature; yellow, high ^206^Pb/^204^Pb). **c**, La/Sm variations across the study region (yellow, high La/Sm—low melt fraction; dark blue, low La/Sm—high melt fraction). **d**, The ^143^Nd/^144^Nd variations across the study region. Yellow indicates a high ^143^Nd/^144^Nd. The topography shown is from the 1 arcsec (∼30 m resolution) Shuttle Radar Topography Mission digital elevation model^48^. Source data

In this Article, we implement a comprehensive sampling strategy, targeting evolutionarily young volcanoes spanning the three rifts (Fig. 1). We analyse rocks that are Quaternary in age (less than 2.58 Myr old) and from volcanoes that have been active during the Holocene, which began 11.7 thousand years ago^21^. By targeting younger rocks, we make a direct comparison with geophysical data across the region, enabling an integrated exploration of mantle petrogenesis and dynamics. Our approach utilizes statistical methods, including semi-parametric regression using splines and K-means cluster analysis to integrate and analyse these geophysical and geochemical data to explore models of upwelling that can explain our data.

## Characteristics of mantle upwellings

Mantle upwellings that originate between depths of 1,000 and 2,800 km are anomalously hot zones and/or zones of an enriched composition that reduce the solidus temperature of the mantle, enabling increased partial melting^22^. Mantle upwellings are widely accepted to contain a variety of domains of differing proportions (for example, high μ (HIMU, U/Pb), enriched mantle I (EMI), enriched mantle II (EMII), common component (C) and Focus Zone (FOZO) (for example, refs. ^5,22–25^)). Such domains typically exhibit an isotopically distinct and enriched composition (generally low ^87^Sr/^86^Sr, high ^143^Nd/^144^Nd and high ^206^Pb/^204^Pb^24^) relative to those of bulk silicate Earth (BSE)^5^. Trace-element ratios such as Ce/Pb and ∆Nb have previously been used to indicate enriched upwellings (>30 (ref. ^26^), >0 (ref. ^27^), respectively) and La/Sm to suggest the melt fraction relative to the study region, with a lower-than-average value suggesting an elevated melt fraction^19^. Mantle upwellings are also commonly associated with reduced (lower) seismic velocities (that is, shear-wave velocity (*v*_s_) and P-wave velocity)^28,29^. These reduced seismic velocities are caused by elevated temperatures and/or the presence of fluids and partial melt^30^.

Crustal assimilation, where crust components are incorporated into the magma, can obscure these geochemical indicators of a deep mantle plume. However, within the Afar region, crustal contamination has played a relatively minor role in recent magmatism^14^ compared with earlier stages of rifting^14,26^ due to the thinning of the present-day crust and because it has been extensively intruded by mafic melts along the length of the rift axes. Seismicity analysis indicates that recent magmatic activity beneath the rift axes in Afar is transient^31^ and, in turn, that magmas are unlikely to reside in crustal reservoirs long enough to extensively assimilate crustal lithologies. Nevertheless, we investigate this issue further in our analysis (‘Probing the presence of mantle upwelling(s) in Afar’).

## Probing the presence of mantle upwelling(s) in Afar

Our study includes over 130 rock samples, with many from previously unstudied volcanoes, roughly doubling the number of high-quality analyses from the area (Fig. 1). The 79 Afar samples included in our study were carefully selected from a repository covering the broader Afar region (details in Methods). These were supplemented by 52 additional samples collected during fieldwork in the MER. To examine spatial trends in the geochemistry of surface volcanism, we analysed all samples for major and trace elements alongside radiogenic isotopes (Sr, Nd, Pb; Methods). We also integrated existing data for 93 rock samples from the open-source GEOROC data repository^32^ (https://georoc.eu/; see Methods for selection criteria), as well as the classic GoA catalogue from ref. ^19^. In addition, we leveraged recent spatial compilations of geophysical variables, such as the depth of the Mohorovičić discontinuity (Moho)^31^ (Methods) and *v*_s_ at regularly spaced depths (40, 60, 80, 100 and 120 km (ref. ^30^)) across the region. These variables provide well-established proxies for the boundary between the crust and mantle and for the presence and abundance of melt within the lithosphere and asthenosphere^30^. Collectively, this information allows us to infer details about the depth, compositional characteristics and relative abundance of partial melts distributed across all three rifts.

**Fig. 2:**
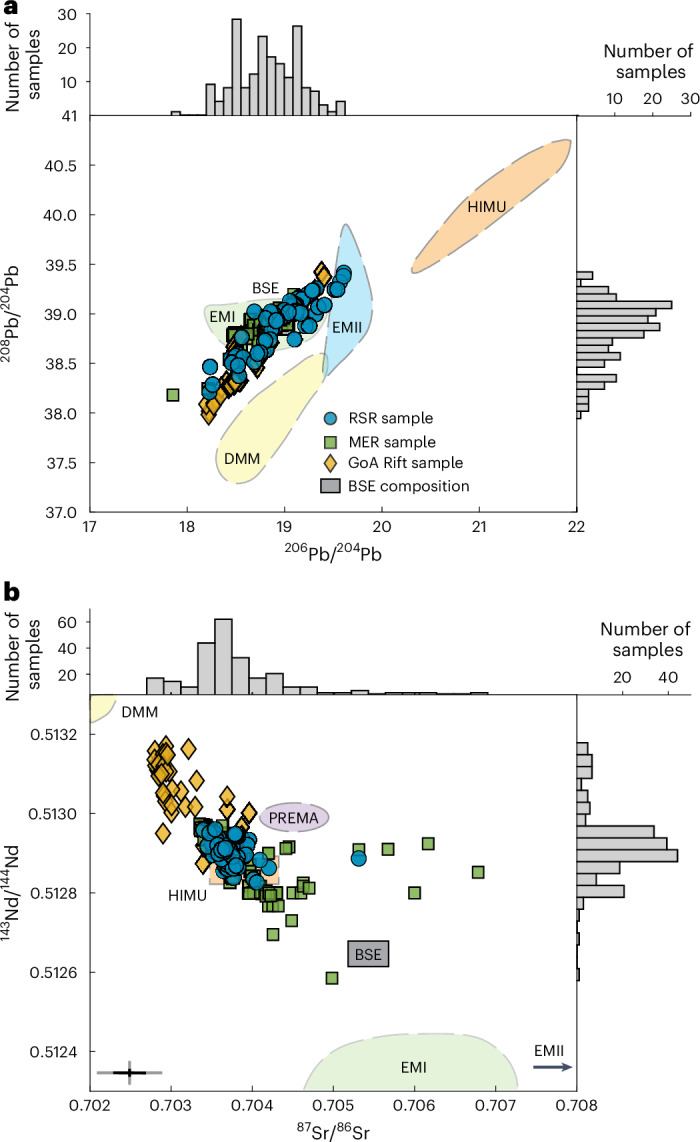
Radiogenic isotope compositions of samples across Afar. **a**, ^206^Pb/^204^Pb versus ^208^Pb/^204^Pb. **b**, ^143^Nd/^144^Nd versus ^87^Sr/^86^Sr. Samples are classified by their rift position, indicated by their symbol colour and shape (RSR, blue circles; MER, green squares; GoA, yellow diamonds). Error bars show the uncertainty associated. Black error bars are the average uncertainty of the dataset; grey are the maximum uncertainty. Uncertainties for data points in **a** are smaller than the symbols. The global mantle endmember compositions (including depleted mid-ocean-ridge-basalt mantle (DMM) and prevalent mantle (PREMA)) are shown as fields behind from refs. ^5,49^. The histograms show the distribution of all data analysed in our study, including our >130 data points.

On the basis of these samples, we infer wide geochemical variability across the study region (Fig. 1). The ∆Nb ranges from −0.26 to 0.94 and the La/Sm ratio ranges from 0.4 to 4.6. The radiogenic isotopes ^206^Pb/^204^Pb, ^207^Pb/^204^Pb, ^208^Pb/^204^Pb, ^87^Sr/^86^Sr and ^143^Nd/^144^Nd also display a large range (Fig. 2 and Extended Data Table 1), with enrichments relative to BSE occurring in all three rifts. Local variability in these radiogenic isotopes is observed within some volcanoes, for example, Boset–Bericha; however, this variability is smaller than the regional range determined for Afar (Fig. 1).

**Extended Data Fig. 1:**
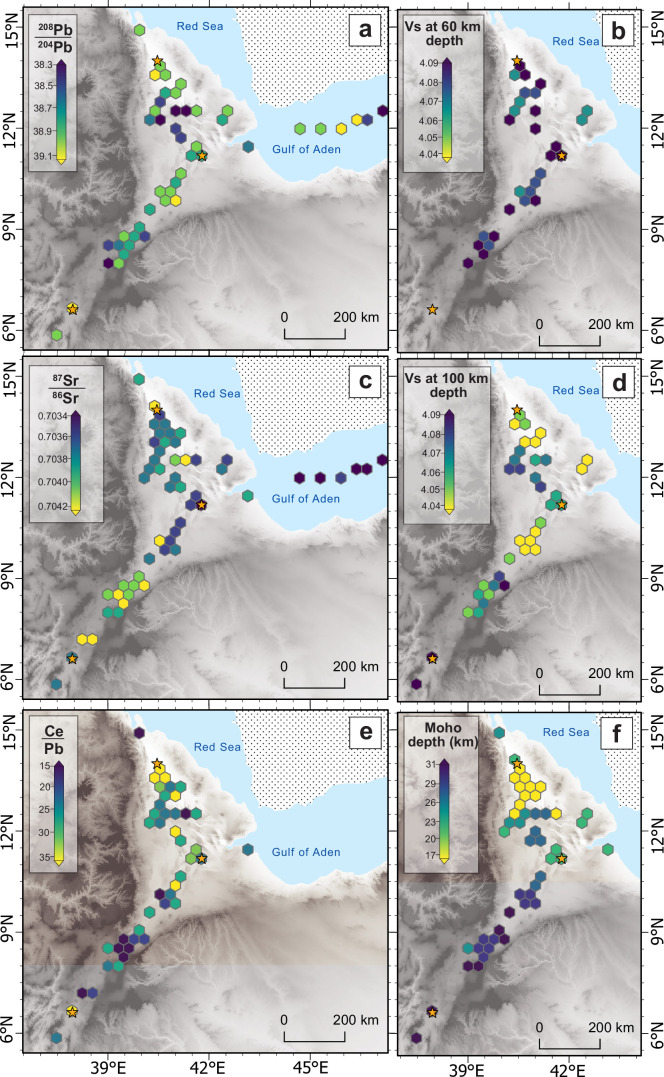
Geochemical and geophysical variations across the study region. Hexmaps showing the patterns for selected variables across the study region (see the main paper and Extended Data Table 2 for further details). **a**, ^208^Pb/^204^Pb of volcanic rocks; **b**, Shear wave velocity (Vs) at 60 km depth; **c**, ^87^Sr/^86^Sr of volcanic rocks; **d**, Shear wave velocity (ν_s_) at 100 km; **e**, Ce/Pb of volcanic rocks; **f**, Moho depth (km). The topography shown is from the 1 arc-sec (∼30 m resolution) Shuttle Radar Topography Mission (SRTM) Digital Elevation Model (DEM)^48^.

Across the study region, the depth of the Moho varies, being shallowest in the RSR (~16 km) and deepest in the MER (31 km). Like the geochemical data, the Vs at 40, 60, 80, 100 and 120 km depths shows regional variability: 3.81–4.05, 4.06–4.17, 4.00–4.15, 3.97–4.10 and 4.02–4.10, respectively (Extended Data Fig. 1). All rifts show zones of high and low Vs (relative to ref. ^30^) in the mantle that vary laterally and in depth.

To evaluate the potential influence of crustal assimilation—which again is considered minor in the Afar region^14^—on mantle composition and upwelling, we assess the correlation between key geochemical and geophysical indicators (see Fig. 4a) and the depth to the Moho. The Moho, the boundary between the crust and mantle, serves as a proxy for crustal thickness, which is widely thought to influence the degree of assimilation^26^ (Methods). We found that most indicators, including Pb isotopes—a reliable indicator of crustal assimilation^33^—exhibit only a weak, but statistically significant, correlation with Moho depth (see Fig. 4a). Further, Ce/Pb exhibits a strong negative correlation (Pearson correlation coefficient of −0.7), indicating that where the crust is thin, the Ce/Pb values are high, and vice versa. This trend can be attributed to minimal crustal assimilation across most of the Afar region, although the degree of assimilation increases as the crust thickens within the MER.

Overall, our dataset shows geochemical and geophysical variability across the study area. The observed variations are consistent with the presence of an upwelling across all three rifts. The spatial trends observed in all variables implicate an underlying complexity to the location of partial melts.

## Models of the Afar upwelling

**Fig. 3:**
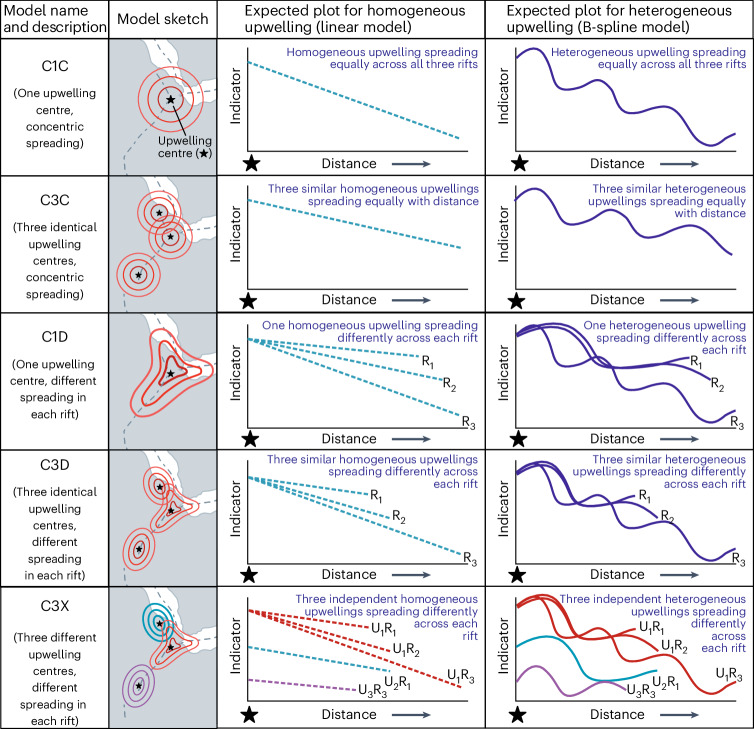
Conceptual models of mantle upwellings beneath Afar tested in this study. The upwelling scenarios for Afar tested in this study. The diagrams (left) are labelled with the code associated with each model (see Extended Data Table 2 and the ‘Statistical models considered’ section within Methods for further details). The locations of the purported mantle upwellings are shown by the star symbol. The number of lines shown on the schematic graphs equals the number of models that must be fitted for that model variant (linear, dashed; spline, continuous). Note that each model variant has been illustrated with an indicator that decreases with a reduction in upwelling proportion. R, rift; U, upwelling.

We used our data to test multiple conceptual models of mantle upwelling dynamics. The initial conceptual model we considered is a simple, homogeneous mantle upwelling at the triple junction (for example, ref. ^19^). This model expects variables (geochemical and geophysical) that indicate deep upwelling to change radially with distance from the upwelling centre (C1C—one centre, circular, concentric; Fig. 3 and Extended Data Table 2). Therefore, this model assumes that variables change linearly from the upwelling centre due to lateral spreading. Extending this model, we then allow the upwelling to be spatially and temporally heterogeneous, as reported for the Hawaiian^34^ and Canary Island^35^ volcanoes. This mechanism yields a similar pattern to the linear C1C model but accommodates compositional fluctuations over the radial distance corresponding to a chemically pulsed upwelling (Fig. 3). This model fits a single spline per parameter for all data against distance from the upwelling centre (spline C1C). The optimum spline allows for regional variations to be accounted for while minimizing noise (optimal smoothing). This approach of both linear and spline fits is applied to all further models described in this section, allowing for homogeneous and heterogeneous upwelling(s), respectively. Note the starting composition of the upwelling is not constrained within the model parameters.

We additionally tested whether the spatial geochemical and geophysical variations observed (Fig. 1 and Extended Data Fig. 1) are best explained through the presence of three small-scale upwellings, which have been proposed on the basis of geophysics and numerical models (for example, ref. ^36^, C3C—three centres, circular, concentric; Fig. 3 and Extended Data Table 1). We tested this model using three upwellings: one centred on the triple junction, one in the northern RSR and one in the southern MER, with the positions of these loci informed by previous models and observations (Methods). This model fits one linear/spline regression per variable (against distance) from the nearest upwelling centre and assumes that the upwellings are compositionally identical and from the same deep source.

It is plausible that the variable tectonic regime (for example, extension rate, crustal thickness) between the three rifts^12^ introduces further complexity to the geochemical and geophysical signals. Accordingly, we introduce three further models, C1D, C3D and C3X (Fig. 3, Extended Data Table 2 and Methods) to account for these regional differences. Models C1D (one centre, different spreading) and C3D (three centres, different spreading) consider one upwelling and three small-scale upwellings, respectively, while allowing for distinct distance-dependent patterns for each rift, thereby modelling the distribution of variables across each rift independently. Unlike the other models, C3X (three independent centres, different spreading) allows each small-scale upwelling to have a distinct signature, as well as permitting an independent distribution along each rift (Methods).

## Spatial characteristics of Afar mantle upwelling

**Fig. 4:**
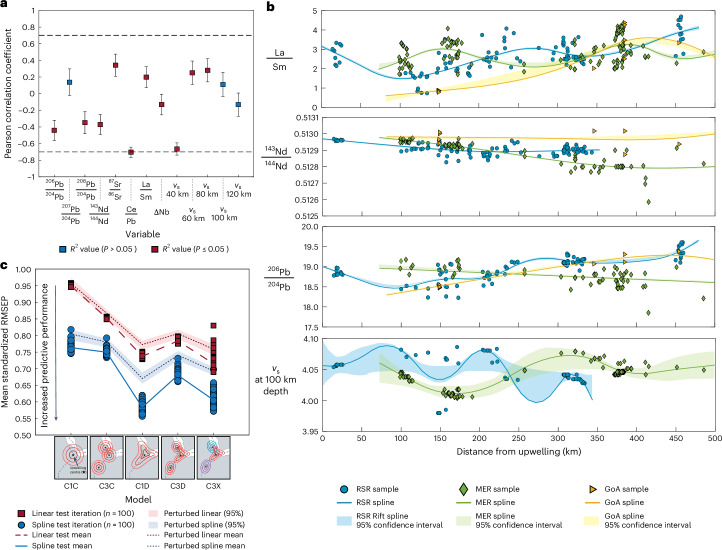
Statistical analysis of rifting models for the RSR, GoA and MER. **a**, Pearson correlation coefficient of each of the selected 13 variables with Moho depth. Error bars show the 95-percentile error of the coefficient (*n* = 250) with the squares representing the mean. Red squares indicate where the correlation is significant (*P* ≤ 0.05), and blue squares indicate that the correlations are not deemed significant (*P* > 0.05). **b**, Splines (a smooth, flexible polynomial curve) of the best overall model—that is, C1C—for selected variables. Symbols show the data within the study (locations denoted by symbols shown); 95% confidence intervals are indicated by the shaded background. The numbers of data observations (*n*) for La/Sm, ^143^Nd/^144^Nd, ^206^Pb/^204^Pb and *v*_s_ at 100 km depth are 269, 218, 185 and 184, respectively. Uncertainties in data values are shown by error bars (average in black, maximum in grey). **c**, The mean standardized RMSEP for each of the models tested. Individual linear model results are shown by red squares, and the mean of those results is displayed by the dashed red line. Individual spline results are shown by blue circles, and the mean of those results is shown by a blue line. All models were run for 100 iterations to capture the probable uncertainty distribution, as shown by the data points. Mean (red and blue dotted lines) and 95% confidence interval (shaded) of results using perturbed data (within the uncertainty of each data point) are also shown (300 perturbation runs each using 100 iterations).

**Extended Data Fig. 2:**
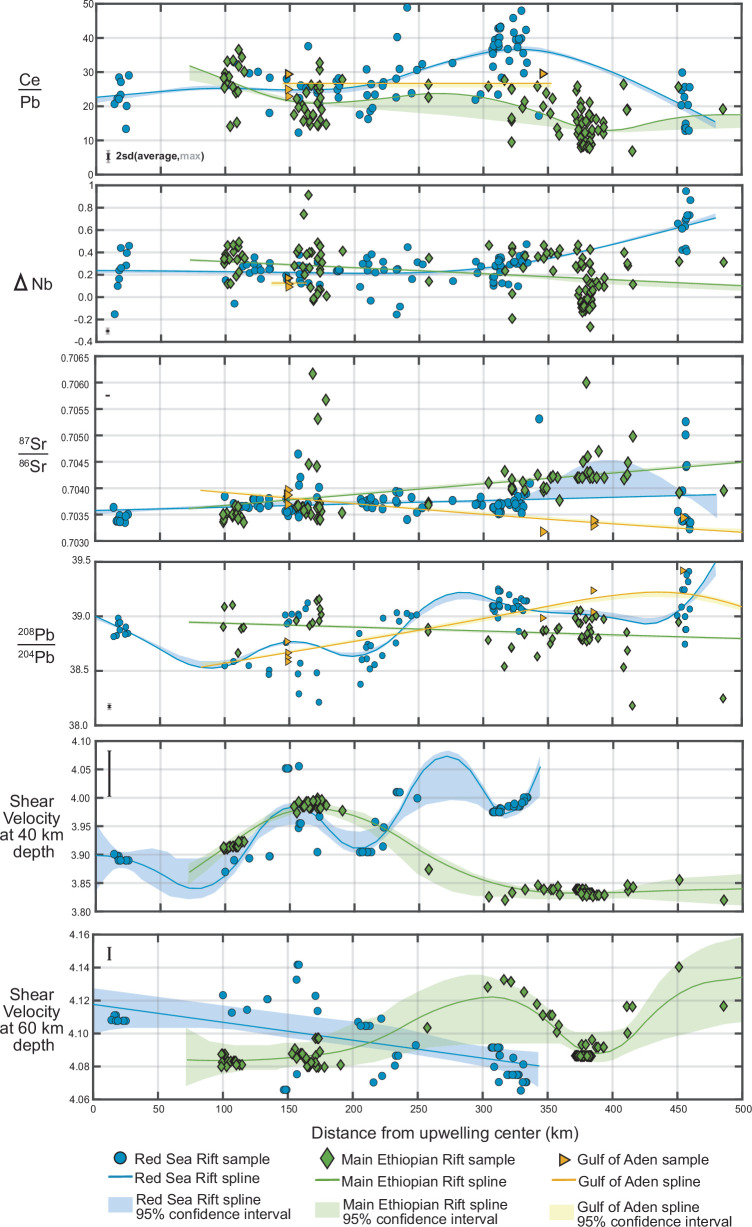
Statistical models of geochemical and geophysical variables. Splines of the winning model (C1C) for remaining selected variables not shown in Fig. 3. Symbols show the data within the study (blue circles = Red Sea Rift, green diamonds = Main Ethiopian Rift, yellow triangles = Gulf of Aden Rift). 95% confidence interval (shaded) of results using perturbed data (within the uncertainty of each datapoint) is also shown (300 perturbation runs each using 100 iterations).

To test these models (Fig. 3 and Extended Data Table 2), we identify 14 key geochemical and geophysical variables (for descriptions, see Extended Data Table 2) and calculate the distance, using the spherical cosine law (Methods), between the purported upwelling centre^15,19,37^ and each observation site (Methods). We then apply two-deep cross-validation (100 iterations) to find the optimum linear fit (representing a homogeneous upwelling) and penalized B-spline fit (representing a heterogeneous upwelling) to each of the variables, using all data points, over a radial distance of 500 km—the radial limit of samples considered within our study (Fig. 4b and Extended Data Fig. 2). The predictive performance of each fit is then assessed by calculating the mean standardized root-mean squared error of prediction (RMSEP), where a value of 1 indicates a lack of predictive capability, and 0 indicates a perfect predictive ability (Fig. 4c).

For all models, we observe the B-spline fit (a class of polynomial function; Methods) to have the best predictive performance, compared with a linear fit (Fig. 4c). This indicates that a compositionally heterogeneous upwelling in Afar is most probable (Fig. 4b,c).

The analysis indicates that the overall best predictive model is the B-spline fit of model C1D, wherein a single, heterogeneous mantle upwelling is present, albeit with differing distributions of geochemical and geophysical variables between rift arms (Fig. 3 and Extended Data Table 2). This model yields a mean standardized RMSEP of 0.59 (Fig. 4c), lower than that of the other models. To further validate our results, we carried out sensitivity analysis, varying the geochemical and geophysical data about their known uncertainties (Methods). The results confirm that model C1D remains the most accurate predictive model (see shaded areas in Fig. 4c).

**Extended Data Fig. 3:**
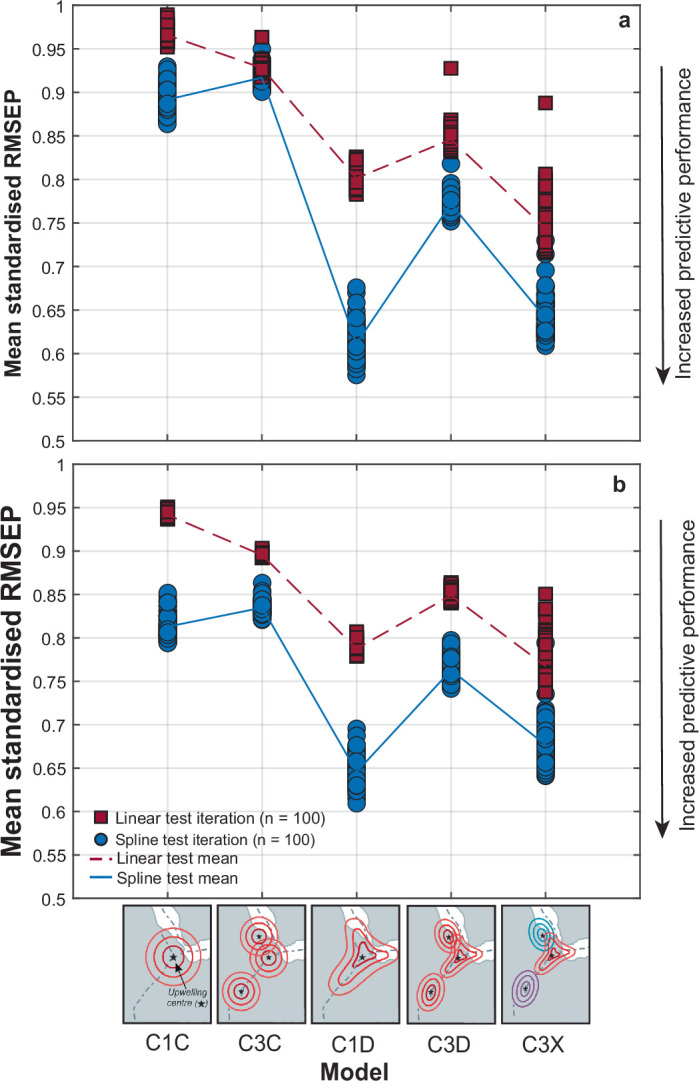
Predictive performance of our statistical models. The mean standardised root means square error of prediction (RMSEP) for each of the models tested (see Extended Data Table 1) when **a**, excluding any observations whereby the analysed rocks have a Ce/Pb < 20, which might indicate some degree of crustal contamination; **b**, excluding all rocks from the Gulf of Aden. In both plots, individual linear model results are shown by red squares and the mean of those results are displayed by the red line. Individual spline results are shown by blue circles and the mean of those results are shown by a blue line. Note that C1D remains the strongest performing model, irrespective of these changes.

While the RSR and MER have a high sample density, there is limited sample availability from the GoA due to poor access. When excluding the GoA from our analyses, the overall trend between the models remains effectively the same (Extended Data Fig. 3b). Although the three rifts share a single, compositionally heterogeneous upwelling, they appear to behave independently, implying that some feature of their tectonic regime modulates the observed signals.

## Interplay between upwelling and segmentation

Many of the optimum splines for each rift display distance-dependent sinusoidal patterns (Fig. 4b and Extended Data Fig. 2). Importantly, our analysis indicates that the variability observed for some variables within the MER exhibits greater amplitude and shorter periodicity with distance from the centre of the upwelling compared with those of the RSR (Fig. 4b and Extended Data Fig. 2). Further, the observed variation in Pb isotopes within the RSR suggests that the upwelling may be chemically heterogeneous for some elements, whereas others show a narrower range in composition (for example, ^87^Sr/^86^Sr is more heterogeneous than ^143^Nd/^144^Nd; Fig. 1 and Extended Data Fig. 1). Although ∆Nb values are almost consistently positive (>0) across the region (except around Boset–Bericha Volcano), we identify small-scale differences in La/Sm and *v*_s_ at 100 km depth, within the likely melt-rich zone of the asthenosphere^26^, with distance to upwelling centre in each rift (Fig. 4b and Extended Data Fig. 2). These small-scale differences indicate locally variable degrees of melting across the study region, agreeing with previous studies that reported low-velocity areas (for example, refs. ^36,38^). This raises the question of whether the zones of locally higher melt fraction, low *v*_s_ and variable geochemistry observed in one rift correspond, spatially and compositionally, to those observed in the other two rifts. If so, this could indicate a shared melt source.

**Fig. 5:**
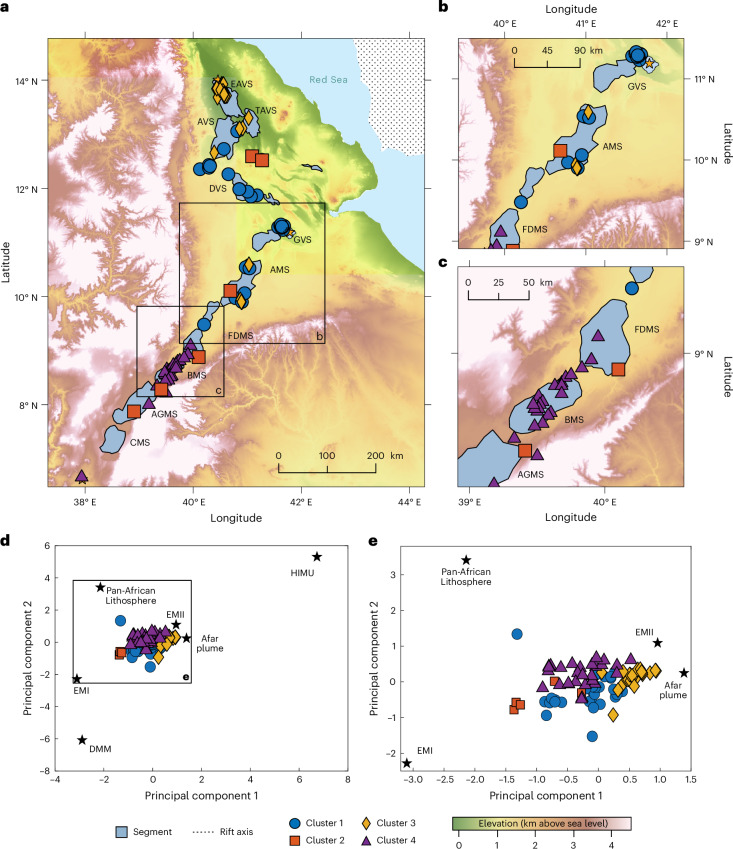
The segments and cluster assignment within the study region. **a**, Segments are shown in blue from north to south: Erta Ale Volcanic Segment (EAVS), Tat’Ale Volcanic Segment (TAVS), Alayta Volcanic Segment (AVS), Dabbahu Volcanic Segment (DVS), Gabillema Volcanic Segment (GVS), Adda’do Magmatic Segment (AMS), Fentale-Dofen Magmatic Segment (FDMS), Boset Magmatic Segment (BMS), Aluto-Gedemsa Magmatic Segment (AGMS) and Corbetti Magmatic Segment (CMS). Rift axis (dotted line) is shown. **b**,**c**, Enlarged maps of the boxes shown in **a**. **d**,**e**, PCA bi-plot (principal component 1 versus principal component 2) when considering the six isotopic systems (Extended Data Table 3) showing the samples and their component scores relative to those of the mantle endmembers. Values used for the mantle endmembers—Pan-African Lithosphere, EMI, EMII, DMM and HIMU—are shown in Extended Data Table 3. The topography shown is from the 1 arcsec (∼30 m resolution) Shuttle Radar Topography Mission digital elevation model^48^.

To address the spatial heterogeneity patterns observed and investigate the potential shared melt source, we carried out principal component analysis (PCA) and K-means cluster analysis using all variables post-standardization (Methods). Across all variables, the K-means cluster analysis algorithm seeks to group similar observations while minimizing the within-cluster total sum of squares for a pre-specified number of clusters. Our K-means cluster analysis shows a higher number of clusters that are smaller in geographic size for the MER (50–100 km length scale; four clusters) compared with the RSR (150–200 km length scale; three clusters) (Fig. 5 and Methods). Several clusters (clusters 1–3) are found to co-exist in different rift arms. For example, samples assigned to cluster 3 are observed in the distal section of the RSR, as well as in locations closer to the MER rift centre (Fig. 5). The three clusters (1–3) observed across the RSR match the initial ~200 km clustering sequence observed across the MER. This sequential repeated clustering may indicate that they are derived from a shared source melt. However, the sequence of these melts—as indicated by clusters—within the MER occurs over a shorter distance compared with that of the RSR.

The spatial distribution of clusters reflects spatial variations in the composition and abundance of melt, which share some cursory similarities to the magmatic segments observed at the surface (Fig. 5). However, when inspecting in detail, we observe clear differences. For example, volcanic systems within both magmatic segments and the adjacent rift flanks are commonly allocated to single clusters, and the boundaries between clusters and known magmatic segments are typically mismatched (Fig. 5). In Afar, the length of the region containing clusters is longer than that of magmatic segments. We therefore infer that the compositional variability of mantle upwelling is unlikely to be related to the along-axis segmentation of crustal subvolcanic plumbing systems.

## Tectonic control on flow of upwelling

Taken together, our data can be explained through a single upwelling model with internal heterogeneity between rifts (for example, refs. ^31,34,39,40^), as shown by the spline model. Crucially, the K-means cluster analysis indicates the signatures of geochemical variability (clusters) are repeated across rifts, implicating pulses of upwelling from the same source, as inferred for other mantle plumes (for example, refs. ^35,39,40^). Rifts act as natural channels for upwelling melt from deeper mantle sources^41^. Considering the high extension rate in the RSR (10.5–19.5 mm yr^–1^ (ref. ^42^)) compared with that of the MER (∼5.2 mm yr^–1^ (ref. ^42^)), it is plausible that a mantle flow rate is impeded by the narrowing of the rift in the MER. This process would lead to a ‘bottleneck’ effect^41,43,44^, which in turn may result in a different length scale of mantle heterogeneity (Fig. 4 and Extended Data Fig. 2) between the RSR and MER (Fig. 5). Further, a contrast in crustal thickness is evident between the rifts, with the MER crust being thicker (25–33 km (ref. ^45^)) than that of RSR (16–25 km (ref. ^46^); Extended Data Fig. 1). Assuming a correlation between crustal and overall plate thickness, this effect is expected to introduce differences in mantle flow rate along each rift in Afar. A progressive thickening of the overlying lithosphere away from the upwelling centre in the MER should reduce the volume capacity for melt, impeding mantle flow. Consequently, the heterogeneous nature of the pulsed upwelling would exhibit a more condensed spatial pattern within the MER compared with RSR, as we observe (Fig. 5).

**Fig. 6:**
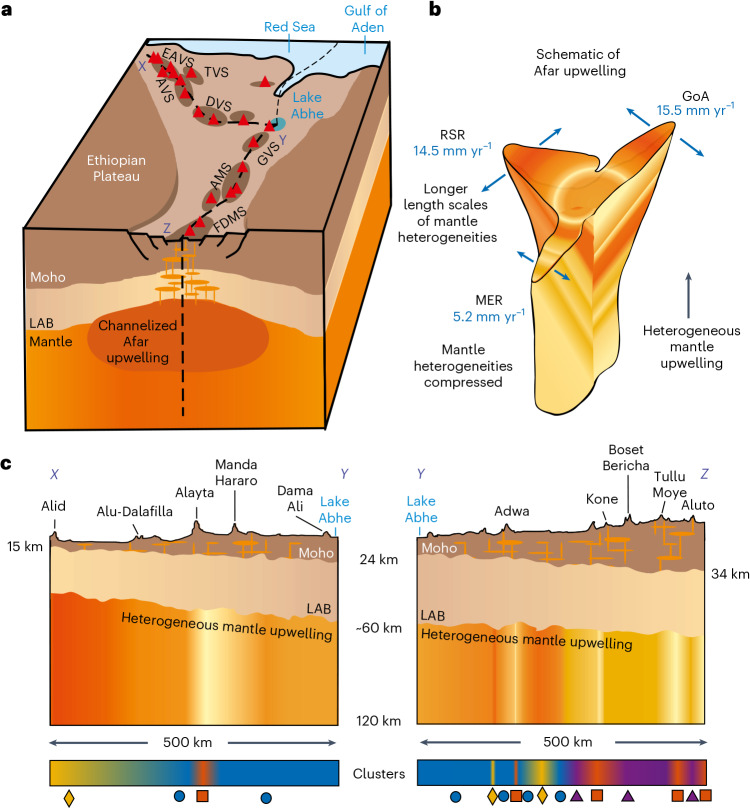
Spatially heterogeneous nature of the mantle upwelling beneath Afar. **a**, The rifts across Afar and the mantle upwelling being channelized by the rift. The lines of section *X*–*Y*–*Z* are those shown in panel **c**. Volcanic segments are shown and labelled. **b**, The Afar upwelling showing the dimensions of channelized flow along the three rifts (dashed lines). **c**, Cross sections along the RSR (line section *X*–*Y*) and MER (*Y*–*Z*) showing the distribution of chemical heterogeneities within the upwelling and how those map to the clusters shown in Fig. 5. Note that the depths of distinct features, including the lithosphere–asthenosphere boundary (LAB), are not shown to scale.

We conclude that variations in melt composition and abundance in and around Afar are best explained by a heterogeneous pulsing mantle upwelling that is not symmetrical (Fig. 5) but is instead shaped by both variable lithospheric thinning and extension rates within each rift (Fig. 6). While this model investigates principally the likelihood of a singular or three small-scale upwelling scenarios, our results demonstrate that for either option, a single heterogeneous upwelling provides the best match to observations in the region. The detected variations in melt composition and abundance between the MER and RSR imply that the length scale of heterogeneities within magma-assisted rifting environments may be controlled not only by the upwelling itself, but also by the extension rate and plate thickness. If this model is correct, it demonstrates that the evolution of a mantle upwelling can be influenced and shaped by the dynamics of the overriding plates.

## Methods

### Sample selection and processing

All samples and previously published data used in this study must originate from a volcano that has been active within the Holocene^21^ (Fig. 1), with the sample estimated to be of Quaternary age (<2.58 Ma). An essential criterion was that all samples have a precisely known location with accurate coordinates.

### Obtaining previously published data

Previously published geochemical data were obtained from GEOROC^19,32^. Once downloaded, the data files were filtered to include only data within Ethiopia (including the MER and Afar). These data were further filtered using the following criteria:

1. The values for the sample must relate to whole-rock geochemistry as opposed to mineral separates.
2. The individual sample must have major-element, trace-element, ^87^Sr/^86^Sr, ^143^Nd/^144^Nd, ^206^Pb/^204^Pb, ^207^Pb/^204^Pb and ^208^Pb/^204^Pb isotope values available.
3. The coordinates must be specific to the individual sample’s location rather than providing an average coordinate for a broader study area.

### Sampling and sample preparation

Ninety-three lavas, 11 welded tuffs and one pumice sample, from various volcanoes in Afar (Erta Ale Volcanic Segment, Ayelu, Abida, Yangudi, Dama Ali, Kerub, Ela, Didoli, Abbahu, Afdera, Tat Ali and Manda Hararo), were selected for geochemical analysis^50–52^. The samples were collected during the CNR/CNRS projects in Afar during the 1960s^53^ and stored in the Afar Repository at the University of Pisa, Italy (http://repositories.dst.unipi.it/index.php/home-afar). A further 52 samples from the Boset–Bericha Volcanic Complex (BBVC) were collected during three field seasons^54^, in November 2012, April–May 2015^55^ and February 2017^56^.

Sample preparation, for major, trace and isotope analyses, was carried out at the University of Southampton. Samples were cut with a saw to remove any weathered sections, and cut surfaces were ground down to reduce any potential contamination by metals from the saw blade. Rock samples were then crushed using a fly press and placed in double-layered plastic bags before crushing to minimize metal contamination during the crushing process.

The crushed material was separated into three size fractions (>1 mm, 0.5–1 mm, <0.5 mm) using Teflon sieves, retaining the middle fraction (0.5–1 mm) for analysis. The selected fraction was cleaned by ultrasonicating in Milli-Q water then dried overnight in an oven at 85 °C. The cleaned rock chips were then hand-picked under a microscope to remove any extraneous (non-rock) material. An aliquot of cleaned chips was used for Pb isotope analysis. For major-element, trace-element, ^143^Nd/^144^Nd and ^87^Sr/^86^Sr isotope analysis, the remaining rock chips were ground to a fine powder using an agate mortar and pestle, again to minimize contamination with metals.

### Trace-element analysis

Samples were prepared for whole-rock trace-element analysis using 0.05 g (for BBVC samples) or 0.075 g (for all other samples) powdered sample. The powdered samples were digested in sealed Savillex Teflon vials with 15 drops concentrated HNO_3_ and 2 ml HF on a hotplate at 130 °C for 24 h (for all samples excluding those from the BBVC) or with 50 drops HF and 0.2 ml HNO_3_ on a hotplate at 130 °C for 24 h (for BBVC samples only). The HNO_3_/HF was evaporated off, and the samples were refluxed in 6 M HCl for another 24 h on a hotplate at 130 °C. The 6 M HCl was evaporated off, and the samples were redissolved in 6 M HCl. Mother solutions were prepared by adding 6 M HCl and Milli-Q water (total 30 ml) to the dissolved samples. Daughter solutions were prepared using 0.5 ml of mother solution, diluted to 5 ml with 3% HNO_3_ (containing the internal standards 5 ppb In/5 ppb Re/20 ppb Be), resulting in an overall dilution factor of ~4,000.

Trace-element analyses of the daughter samples were undertaken on the Thermo Scientific X Series 2 quadrupole inductively coupled plasma mass spectrometer (ICP-MS) at the University of Southampton. Samples and standards were spiked with internal standard elements and corrected for interferences and the blank and then calibrated using a suite of international rock standards. Accuracy was monitored using reference materials JA-2, BCR-2 and JB-2 (Supplementary Tables 1 and 2).

### Pb isotopic analysis

For Pb isotope analysis, 0.3 g of cleaned, picked rock chips (0.5–1.0 mm) were weighed into Pb Savillex Teflon vials and leached on a hotplate with 4 ml 6 M HCl for 1 h (15 min for obsidian and pumice samples, to avoid full dissolution of the sample). Samples were rinsed several times in Milli-Q water then 0.5 ml concentrated HNO_3_ before adding 3–4 ml of concentrated HF. Samples were digested, following the same procedure as for trace elements, and refluxed on a hotplate at 130 °C for 24 h, before being evaporated to dryness. Then 0.5 ml concentrated HCl was added, and the sample was evaporated to dryness. Then 0.5 ml concentrated HNO_3_ was added and again evaporated to dryness. The final residue was reconstituted in 0.5 ml HBr and refluxed for 1 h. The samples were cooled and centrifuged for 5 min. Pb was isolated using a single-stage HCl anion-exchange chromatographic resin separation method^57^, with AGX-1×8, 200–400 mesh resin. Following this, the Pb isolate was dried down, redissolved in HNO_3_ and analysed using the double-spike method of ref. ^58^. The samples were subsequently analysed on a Thermo Scientific Neptune multi-collector inductively coupled plasma mass spectrometer (MC-ICP-MS) at the University of Southampton (UK), achieving an NBS SRM 981 reproducibility of ^206^Pb/^204^Pb = 16.9404 ± 24 (142 ppm), ^207^Pb/^204^Pb = 15.4969 ± 26 (168 ppm), ^208^Pb/^204^Pb = 36.7149 ± 66 (180 ppm) (2 s.d.; *n* = 44). Pb isotope measurements of the standard are within error of the accepted values (^206^Pb/^204^Pb = 16.9412, ^207^Pb/^204^Pb = 15.4988, ^208^Pb/^204^Pb = 36.7233). Accuracy was 47 ppm for ^206^Pb/^204^Pb, 123 ppm for ^207^Pb/^204^Pb and 174 ppm for ^208^Pb/^204^Pb.

### ^143^Nd/^144^Nd and ^87^Sr/^86^Sr isotopic analysis

For Sr and Nd analysis, the remaining mother solutions from the preparation of trace-element solutions (see the preceding) were used for all samples except those of the BBVC. An aliquot of each mother solution was used to give a volume of liquid containing at least 1 μg Sr and 200 ng Nd and evaporated to dryness in Savillex Teflon vials on a hotplate at 130 °C. Sample residues were reconstituted in 200 μl 1.75 M HCl. For the BBVC samples, rock chips were leached in 4 ml 6 M HCl for 30 min in Savillex Teflon vials (obsidian samples for only 15 min to avoid full dissolution of the sample). The samples were then rinsed with Milli-Q water and HNO_3_, and then the same digestion procedure as for the preceding trace-element analysis was followed. The final mother solutions were prepared using HCl and Milli-Q water to 30 ml for felsic samples and 20 ml for mafic samples.

All samples were then passed through ion-exchange column chemistry using an AG50-X8 200–400 mesh resin cation column to separate the Sr and Nd fractions. The sample fractions were subsequently evaporated to dryness, ready for further column chemistry.

Sr was further isolated through Sr-spec resin columns following the methodology of ref. ^59^. Samples were then evaporated to dryness, dissolved in 1.5 ml 1 M HCl and loaded onto outgassed tantalum filaments with 1 μl of Ta-activator. Sr isotopic analysis was performed on a thermal ionization mass spectrometer Thermo Scientific Triton Plus at the University of Southampton. Reference material SRM NIST987 (^87^Sr/^86^Sr = 0.710258; GeoREM) was used to monitor accuracy and gave average ^87^Sr/^86^Sr values of 0.710243. All samples were normalized to NBS SRM-987 ^87^Sr/^86^Sr = 0.710248 (ref. ^60^), while reproducibility was ±0.000020 (28.2 ppm, 2 s.d.; *n* = 464). Accuracy was 21 ppm.

The Nd aliquot from the cation column was followed by an Ln-spec resin (50–100 μm) (ref. ^59^). The samples were then evaporated to dryness and 3% HNO_3_ was added to produce a solution of 50 ppb. ^143^Nd/^144^Nd analyses were undertaken on the ThermoScientific Neptune MC-ICP-MS at the University of Southampton. Corrected Nd isotopic compositions were obtained using a method based on ref. ^61^ through adjustment to a ^146^Nd/^144^Nd ratio of 0.7219 and a secondary normalization to ^142^Nd/^144^Nd = 1.141876. Reference material JNdi-1 was measured as an unknown (^143^Nd/^144^Nd of 0.512124, 2 s.d. (refs. ^62,63^)), achieving an average ^143^Nd/^144^Nd of 0.512115 with an external reproducibility of ±0.000008 (2 s.d., 15.2 ppm) across six analysis sessions over 2 years. The total column blanks (when blank acid is run through the column procedure) were negligible (<20 pg) compared with the total amounts analysed (1 μg and 200 μg) for Sr and Nd, respectively.

### *v*_s_ mapping from joint inversion

We use the *v*_s_ model of ref. ^30^ for inclusion in our analysis. The three-dimensional velocity model is created through a joint inversion of Rayleigh-wave phase velocities from ambient noise and teleseisms^30,64^. The *v*_s_ model is parameterized every 5 km vertically with 0.1° × 0.1° pixel size for the upper 50 km. For deeper depths, an irregular spacing was used, increasing from 10 to 50 km spacings to match that of ref. ^38^. For further details on the creation of the velocity model, the reader is directed to refs. ^30,64^ and references therein.

For the analysis in this Article, the *v*_s_ model was interpolated to 1 km depth using a linear interpolation; we then extracted one-dimensional columns of velocity with depth at the same resolution as our pixel size (0.1° × 0.1°).

### Moho depths

The gridded Moho depth map was produced from the *v*_s_ maps of ref. ^64^, described in the preceding. The *v*_s_ model was interpolated to a vertical grid spacing of 1 km. A velocity slice at the 3.75 km s^–1^ contour was extracted, which mapped best to previous receiver function measurements^65–69^, active source experiments (for example, ref. ^45^) and previous S-wave models (for example, ref. ^70^).

### Statistical models considered

As described in the text, five models were considered (Extended Data Table 2), with each model being tested using a linear fit and a spline fit (Fig. 3). We note that a spline fit itself can be linear if that is the best-fitting line.

Empirical models are estimated for the variation of each of 14 geochemical quantities (each of which is represented generically by random variable *Y*) as a function of distance *d* ∈ [0, 1,800] km for the five different models. Models are specified that explore the variation of *Y* with *d* in increasing complexity. The simplest model (C1C) assumes the existence of a single upwelling centre (at 11.192° N, 41.784° E; Figs. 1 and 3), with respect to which *d* is defined for all three rifts. The variation of *Y* with *d* is assumed common to all rifts. Model C3C assumes the existence of three upwelling centres (at 11.192° N, 41.784° E; 14.008° N, 40.458° E; and 6.626° N, 37.948° E; Fig. 1) on the basis of ref. ^36^; observations are allocated to the nearest upwelling centre, facilitating calculation of a single *d* for each observation. Like model C1C, the variation of *Y* with *d* is assumed common to all rifts, regardless of upwelling allocation. Model C1D assumes one upwelling centre (like C1C) for calculation of *d*, but now the variation of *Y* with *d* is assumed to be different across rifts. Model C3D duplicates C3C for estimation of *d*, but variation of *Y* with *d* is assumed to be different across rifts. Finally, in model C3X, we consider the presence of three upwelling centres, with different variation of *Y* with *d* for each combination of upwelling and rift.

### Data pre-processing

For models C1C and C1D, the distance between each sample and the upwelling locus centred on Lake Abhe (11.192170° N, 41.783750° E) is calculated. For models C3C, C3D and C3X, the distance between each sample and each of the three upwelling locations (Figs. 1 and 3) is measured, and then each sample is assigned to its nearest upwelling centre. The distance (*d*) between two locations (upwelling and sample) is calculated using the spherical cosine law:

1 $$d=R\left({\cos }^{-1}\left(\cos \left(a\right)\cos \left(b\right)+\sin \left(a\right)\sin \left(b\right)\cos \left(C\,\right)\right)\right.$$

where *a* is the angle (in radians) from the North Pole to the sample location, *b* is the angle (in radians) from the North Pole to the upwelling location, *C* is the difference (in radians) between the longitude values of the sample and upwelling, and *R* is the radius of the Earth in metres (6,371 × 10^3^).

### Penalized B-splines

For each model, the variation of *Y* with *d* is described using a penalized B-spline (for example, refs. ^71,72^), the characteristics of which are selected to provide optimal predictive performance. First, for a large index set of locations equally spaced on the domain of distance, we calculate a B-spline basis matrix, *B* (for example, ref. ^73^) consisting of *p* equally spaced cubic spline basis functions. Then the value of *Y* on the index set is given by the vector *B***β** for spline coefficient vector **β** to be estimated. The value of *p* is specified to be sufficiently large to provide a good description of a highly variable *Y*. For a given dataset, we penalize the difference between consecutive values in **β** using a roughness penalty, such that the penalized spline exhibits optimal roughness, providing optimal predictive performance.

### Estimating optimal spline roughness and predictive performance

For a sample of *n*_1_ training data, consisting of vectors of geochemical and geophysical quantities (**y**_1_) and distances (**d**_1_), we first allocate each element of **d**_1_ to its nearest neighbour in the index set and hence construct the appropriate spline basis matrix *B*_1_ for the sample. We then assume that ${\bf{y}_{1}}={B}_{1}\bf{\upbeta} +\bf{\upepsilon}$, where the elements of **ε** are independently and identically distributed zero-mean Gaussian random variables. We penalize the roughness of **β** using a first-different penalty *λ***β'***P***β**, where $P=D{^\prime} D$ and *D* is a first-difference matrix (with elements ${D}_{{ij}}=-1$ if $i=j$; $=1$ if $j=i+1$; and $=0$ otherwise (for example, ref. ^74^). For a given choice of $\lambda$, we then find the optimal value of **β** by minimizing lack of fit:

2 $${\mathbf{\upbeta}}^{* }\left(\lambda \right)=\begin{array}{l}\displaystyle {{\mathrm{argmin}}}\,\{\left(\bf{y}_{1}-{B}_{1}{\mathbf{\upbeta}} \right){^\prime} \left(\bf{y}_{1}-{B}_{1}{\mathbf{\upbeta}} \right)+\lambda \beta {^\prime} P{\mathbf{\upbeta}} \}\\ \displaystyle \quad{\mathbf{\upbeta}} \atop \end{array}$$

3 $$={({B}_{1}^{{\prime} }{B}_{1}+\lambda P)}^{-1}{B}_{1}^{{\prime} }{\bf{y}}_{1}$$

We can evaluate the predictive performance of the resulting spline description using a tuning set of *n*_2_ observations (independent of the training set) represented by vectors **y**_2_ and **d**_2_. We again start by finding the appropriate spline basis matrix *B*_2_ for this sample. Then we can calculate the predictive mean square error for the tuning sample:

4 $${\text{MSE}}_{{{\mathrm{Tune}}}}\left({\rm{\lambda }}\right)=\frac{1}{{n}_{2}}{\left(\bf{y}_{2}-{B}_{2}{\mathbf{\upbeta}}^{* }\left({\rm{\lambda }}\right)\right)}^{{\prime} }\left(\bf{y}_{2}-{B}_{2}{\mathbf{\upbeta} }^{* }\left({\rm{\lambda }}\right)\right)$$

for each of a set of representative choices of values for *λ*. We can then select the optimal value of *λ* using

5 $${\lambda }^{* }=\begin{array}{l} \displaystyle {{\mathrm{argmin}}}\;\{{\text{MSE}}_{{{\mathrm{Tune}}}}\left(\lambda \right)\}\\ \displaystyle \quad \lambda \atop \end{array}$$

The value ${\text{MSE}}_{{{\mathrm{Tune}}}}\left({\lambda }^{* }\right)$ is a biased estimate of predictive performance since the value of ${\lambda }^{* }$ was tuned to minimize its value. We can obtain an unbiased estimate for the predictive performance of the spline model using a test set of *n*_3_ observations (independent of the training and tuning sets) represented by vectors **y**_3_ and **d**_3_ (and corresponding spline basis matrix *B*_3_). Then the predictive performance is estimated using:

6 $${{{\mathrm{MSE}}}}_{{{\mathrm{Test}}}}=\frac{1}{{n}_{3}}{\left(\bf{y}_{3}-{B}_{3}{\mathbf{\upbeta}}^{* }\left({{\rm{\lambda }}}^{* }\right)\right)}^{{\prime} }\left(\bf{y}_{3}-{B}_{3}{\mathbf{\upbeta}}^{* }\left({{\rm{\lambda }}}^{* }\right)\right)$$

### Cross-validation and model comparison

We exploit cross-validation to evaluate MSE_Test_ by partitioning the full sample of data into *k* > 2 groups at random, withholding one group for tuning and another group for testing and retaining the remaining *k* – 2 groups for training. We then loop exhaustively over all possible combinations of choice of train, tune and test groups, evaluating overall predictive performance on the test data over all iterations, noting that each observation occurs exactly once in the test set. For models requiring separate model fits to subsets of data (C1D, C3D, C3X), MSE_Test_ is estimated using predictions from optimal predictive models for each subset. Further, we can repeat the analysis for different initial random partitioning of observations into *k* groups to assess the sensitivity of overall predictive performance to this choice. We are careful to use the same cross-validation partitions to evaluate each of the five models so that predictive performances can be compared fairly.

To quantify model performance over all 14 geochemical quantities $(j=\mathrm{1,2},\ldots ,13)$, we define the overall standardized MSE_Test_

7 $$\text{SMSE}=\mathop{\sum}\limits_{j=1}^{13}\frac{{\text{MSE}}_{{{\mathrm{Test}}},\,j}}{{s}_{j}^{2}}$$

where MSE_Test,*j*_ is the predictive performance for the *j*th geochemical indicator, and $\,{s}_{j}^{2}$ is the sample estimate for the variance of that quantity. The estimation of the splines and the testing of their predictive performance was repeated over 100 iterations. Results from each iteration and the mean of the SMSE are shown in Fig. 4.

### Linear regression

For comparison, we also evaluate linear regression models for the variation of *Y* with *d*. In the current notation, these can be thought of as simple models with basis matrix *B* = [**1** **d**], where **1** is a vector of appropriate length with each element = 1. *Β* in this case is a 2-vector with elements corresponding to intercept and slope coefficients. Linear regression is approached using penalized B-spline models as the roughness coefficient *λ* → *∞*. That is, linear regression corresponds to a penalized B-spline model with very large *λ*. Therefore, a penalized B-spline model is guaranteed to perform at least as well as linear regression.

### Uncertainty of model performance

To explore the effect of uncertainty on model performance, a perturbation analysis was undertaken. This analysis required the generation and modelling of *n*_Pert_ new data samples. Each of these data samples corresponded to a perturbation of the original data sample. A value of *n*_Pert_ = 300 was selected to ensure that 95% uncertainty bands for predictive performance on perturbed data could be estimated with confidence.

In perturbed sample *q*, ${q=1,2,\ldots ,n}_{{{\mathrm{Pert}}}}$, the value ${y}_{{ijq}}^{* }$ of the *i*th observation for variable *j* was obtained by perturbing the corresponding value ${y}_{{ij}}$ in the original data sample, using additive Gaussian noise ${e}_{{ijq}}$, the standard deviation ${\sigma }_{{ij}}$ of which was informed by the known value of measurement uncertainty for that observation of the variable. Mathematically:

8 $${y}_{{ijq}}^{* }={y}_{{ij}}+{e}_{{ijq}}$$

The complete modelling procedure was then applied to each perturbed data sample in turn. The predictive performance of different models was assessed over the *n*_Pert_ perturbations, as illustrated in Fig. 4, in terms of 95% uncertainty bands. The figure indicates that model C1D provides the best predictive performance on perturbed data, as well as for the original unperturbed sample. Note that, since noise has been added to observations in the perturbation analysis, the overall performance of models on perturbed data is poorer than on the original sample, as expected.

### Testing the influence of crustal assimilation

We tested the influence of crustal assimilation further by excluding cases where Ce/Pb values fall below 20 and which could feasibly be associated with crustal assimilation^26,33^. Using additional analysis, we confirm that excluding cases in which Ce/Pb < 20 does not affect our overall results (Extended Data Fig. 3a), suggesting that primary mantle compositional fluctuations (relative proportions of compositional mantle endmembers) exert the first-order control on eruptive compositions at the surface.

### PCA

PCA requires each sample or object to have the same number of values for each variable, so the dataset was reduced to 94 samples. PCA is carried out only on radiogenic isotope compositions of the samples where data are available for the mantle endmembers investigated (Afar plume, Pan-African Lithosphere, Depleted Mantle, EMI, EMII, HIMU; Fig. 5 and Extended Data Table 3; ref. ^75^). While other purely geochemical studies on Afar (for example, refs. ^14,37^) have included sub-crustal components such as the sub-continental lithospheric mantle, we decided not to include this endmember as it can sometimes be indistinguishable from certain mantle endmembers (EM1), especially in cases where the sub-continental lithospheric mantle is metasomatized. The preferred values used for the endmembers in our models are provided in Extended Data Table 3. Each object is standardized before being included in the PCA:

9 $${y}_{{\text{std}}j}=\frac{{{y}_{\!j}}-{{\bar{y}}_{\!j}}}{{s}_{\!j}}$$

where ${\bar{y}}_{j}$ is the mean of variable *j*, and *s*_*j*_ is the standard deviation of the variable *j*:

10 $${{s}_{\!j}}=\sqrt{\frac{\sum {\left({{y}_{\!j}}-{{\bar{y}}_{\!j}}\right)}^{2}}{{N}_{\!j}}}$$

where *N*_*j*_ is the number of objects within variable *j*.

Approximately 90.5% of the variance is explained within the plane of the first two eigenvectors, increasing to 95.5% when including the third eigenvector. The first principal component (PC-1) is most influenced by ^207^Pb/^204^Pb and ^208^Pb/^204^Pb, whereas the second principal component (PC-2) is dominantly influenced by ^206^Pb/^204^Pb and ^87^Sr/^86^Sr. The third principal component (PC-3) is dominated by ^207^Pb/^204^Pb and ^143^Nd/^144^Nd (Supplementary Table 3).

### K-means cluster analysis

K-means cluster analysis^76^ was carried out on the samples using the 13 standardized variables (excluding Moho depth; Extended Data Table 1; refs. ^77–80,75^). The K-means algorithm assigns each object to a singular cluster that does not overlap with another (partitional clustering), minimizing the total sum of squared errors from the centre point of each cluster, known as the centroid, to each data point.

To find the optimum number of clusters (*k*)—which reduces the within-cluster total sum of squared errors with the lowest number of clusters—we run the K-means algorithm specifying *k* to be 1/20 over 1,000 iterations for each *k* (Supplementary Fig. 1). We then select four clusters on the basis of *k* = 4, reducing the within-cluster total sum of squares by 60% from *k* = 1 and the range over the 1,000 iterations being minimized when *k* ≥ 4. The cluster assignments for each object, out of the 1,000 iterations, are selected by finding the iteration number that is closest to the mean within-cluster total sum of squares of that *k* value (shown by the blue line in Supplementary Fig. 1).

## Online content

Any methods, additional references, Nature Portfolio reporting summaries, source data, extended data, supplementary information, acknowledgements, peer review information; details of author contributions and competing interests; and statements of data and code availability are available at 10.1038/s41561-025-01717-0.

## Supplementary information

Supplementary Information Supplementary Figs. 1 and 2 and Tables 1–3.

## Source data

Source Data Fig. 1 Data used in the study, statistical source data.

## Figures and Tables

**Extended Data Table 1:**
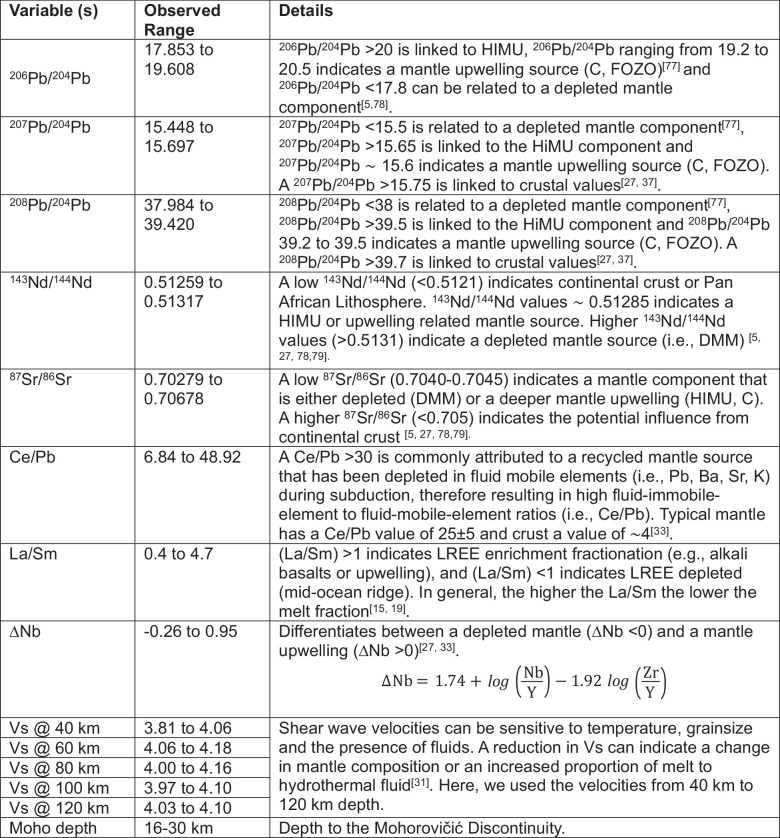
Variables used within the analysis

Variables used within the analysis summarising the ranges observed and justifying their selection based on arguments made in prior work.

**Extended Data Table 2:**
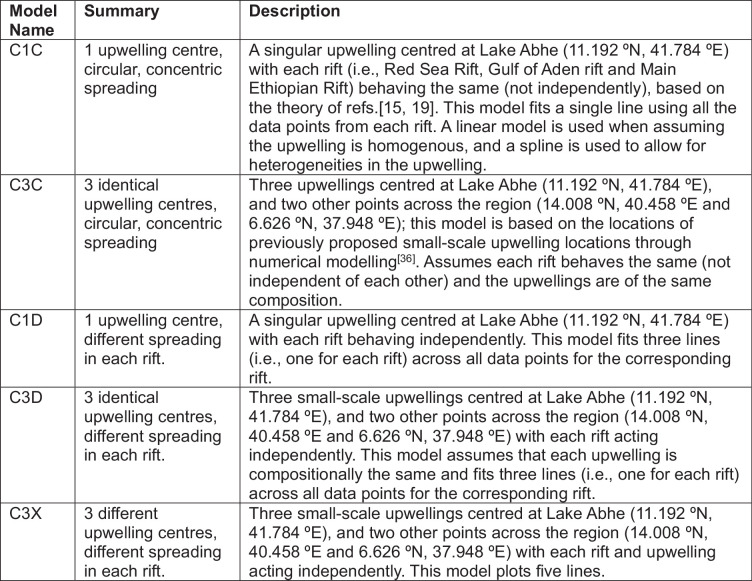
Description of models considered

Alternative models considered when assessing the upwelling characteristics in Afar (see Fig. 3 for visual representation). Note that each of these five models is further modelled twice, using a linear fit (representing a homogenous upwelling), and a spline fit (representing a heterogenous upwelling), giving ten model permutations.

**Extended Data Table 3:**
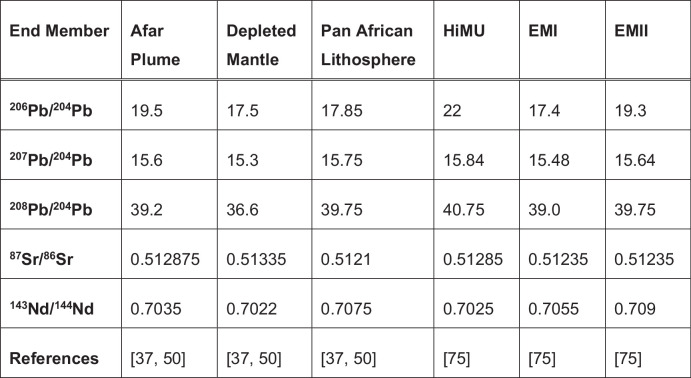
Mantle and crustal end-member compositions

Mantle and crustal end-member compositions used in the principal component analysis.

## Data Availability

The datasets analysed for the current project are available as Supplementary Information. Some data were obtained from GEOROC^19,32,50^; these data are clearly marked in the datafile. The data are freely available via figshare at 10.6084/m9.figshare.28769105 (ref. ^81^). Source data are provided with this paper.

## Code availability

The input data, code and output within this study are openly available via GitHub at https://github.com/ygraigarw/AfarPlume.git (ref. ^82^).
