# Supplementary material for: Mantle upwelling at Afar triple junction shaped by overriding plate dynamics
Source: Nat Geosci. 2025 Jun 25;18(7):661–9. doi: 10.1038/s41561-025-01717-0 (PMC12245717; doi:10.1038/s41561-025-01717-0)
Supplement: Supplementary file 1 — Supplementary Figs. 1 and 2 and Tables 1–3. [file 41561_2025_1717_MOESM1_ESM.pdf]

nature geoscience

Supplementary information https://doi.org/10.1038/s41561-025-01717-0

Mantle upwelling at Afar triple junction shaped by overriding plate dynamics

In the format provided by the authors and unedited

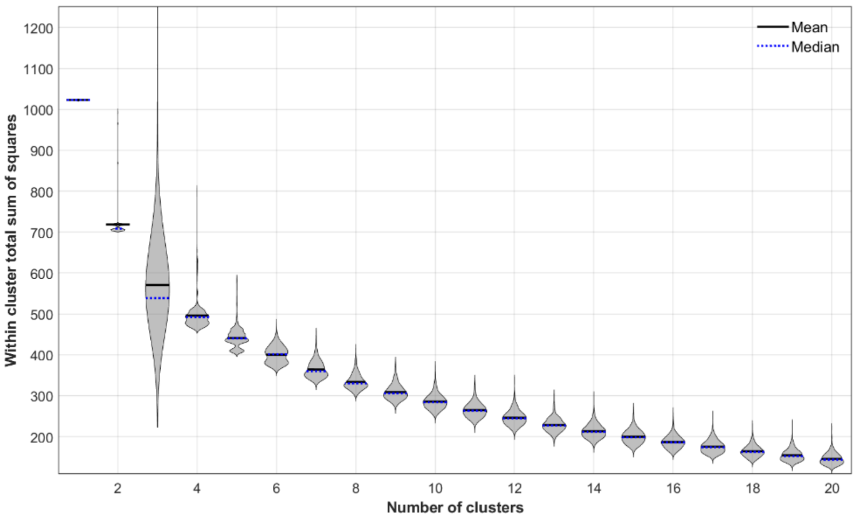

Supplementary Fig. 1: Results of k-means cluster analysis. Violin plot showing the within cluster sum of squares for the k–means cluster analysis testing number of clusters between 1 and 20, for 1000 iterations. Number of observations, n = 95.

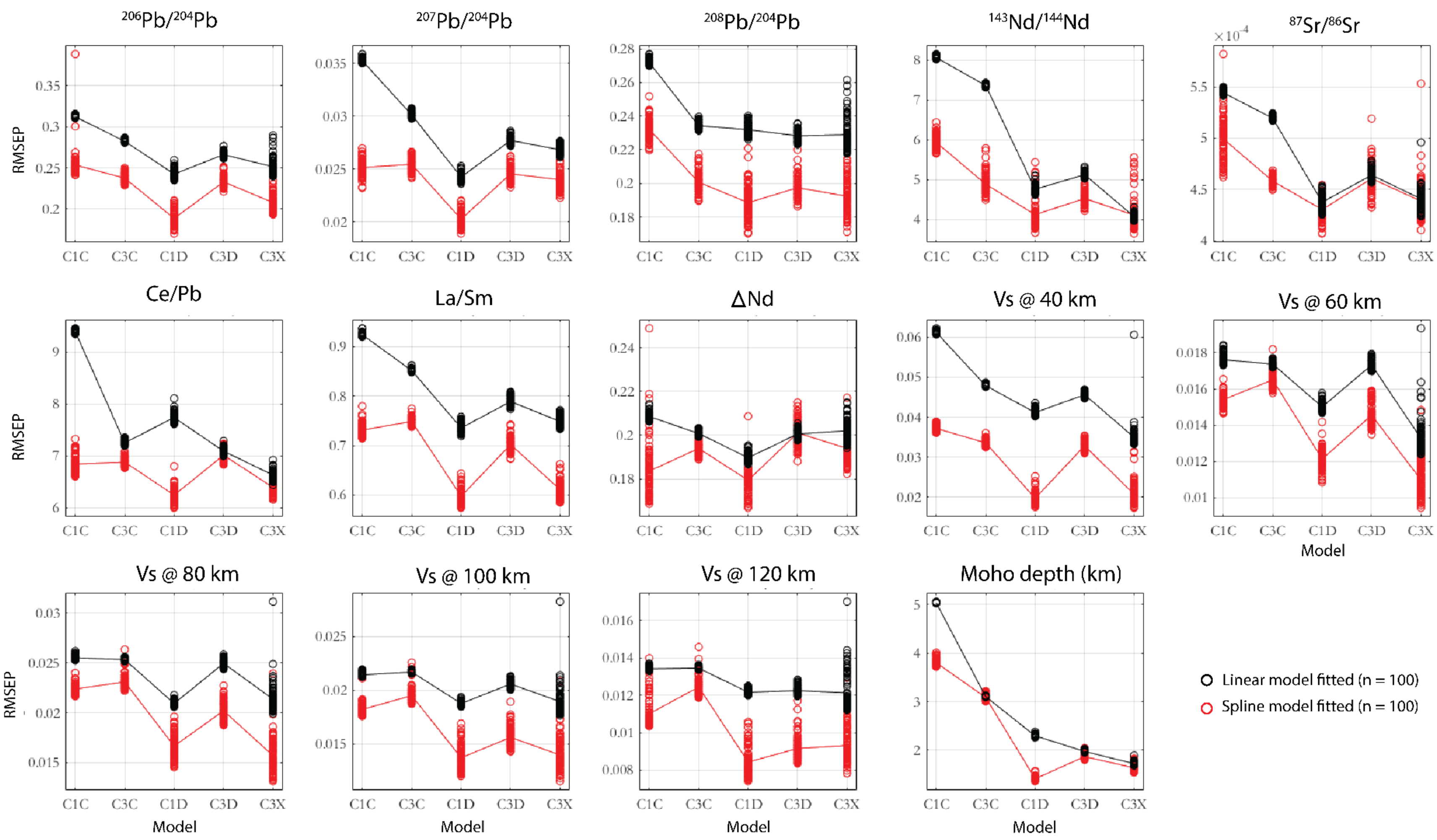

Supplementary Fig. 2: Model results for each variable. RMSEP results for each variable showing the performance of the linear (black) and spline (red) fit. The lower the RMSEP, the better the predictive performance.

	JA-2 (Imai et al., 1995), n=6					BCR-2 (Wilson., 1997), n=4				
	Mean (ppm)	Std.Dev	%RSD	Ref.	Uncert.	Mean (ppm)	Std.Dev	%RSD	Ref.	Uncert.
**Li**	29.41	0.25	0.85	29.18	0.60	9.04	0.12	1.33	9.13	0.22
**Sc**	17.91	0.19	1.06	18.93	0.30	32.55	0.17	0.52	33.53	0.40
**V**	115.38	4.32	3.74	119.70	2.40	403.50	5.06	1.25	417.60	4.50
**Cr**	397.18	13.36	3.36	424.80	9.30	14.56	0.47	3.23	15.85	0.38
**Co**	27.55	0.20	0.73	28.33	1.00	36.86	0.19	0.52	37.33	0.37
**Ni**	127.20	1.68	1.32	136.00	2.20	11.72	0.41	3.50	12.57	0.30
**Cu**	30.36	0.97	3.19	29.00	1.50	23.06	1.24	5.38	19.66	0.72
**Rb**	72.17	1.29	1.79	69.80	1.30	46.46	0.92	1.98	46.02	0.56
**Sr**	245.67	1.43	0.58	245.80	3.00	333.78	1.47	0.44	337.40	6.70
**Y**	17.17	0.07	0.41	16.89	0.60	36.01	0.09	0.25	36.07	0.37
**Zr**	114.72	0.75	0.65	108.50	2.60	187.65	1.74	0.93	186.50	1.50
**Nb**	9.24	0.10	1.08	9.30	0.20	12.43	0.24	1.93	12.44	0.20
**Cs**	4.97	0.11	2.21	4.78	0.10	1.11	0.04	3.60	1.16	0.13
**Ba**	317.67	5.45	1.72	308.40	5.10	692.20	12.06	1.74	683.90	4.70
**La**	16.08	0.05	0.31	15.46	0.40	24.92	0.16	0.64	25.08	0.16
**Ce**	33.16	0.25	0.75	32.86	0.90	52.85	0.29	0.55	53.12	0.33
**Pr**	3.77	0.02	0.53	3.69	0.10	6.79	0.05	0.74	6.83	0.04
**Nd**	14.37	0.07	0.49	14.04	0.20	28.41	0.10	0.35	28.26	0.37
**Sm**	3.08	0.02	0.65	3.03	0.00	6.54	0.05	0.76	6.55	0.05
**Eu**	0.91	0.01	1.10	0.89	0.00	1.97	0.02	1.02	1.99	0.02
**Gd**	3.04	0.04	1.32	3.01	0.10	6.70	0.08	1.19	6.81	0.08
**Tb**	0.49	0.01	2.04	0.48	0.00	1.06	0.01	0.94	1.08	0.03
**Dy**	2.93	0.03	1.02	2.85	0.10	6.36	0.05	0.79	6.42	0.06
**Ho**	0.60	0.00	0.00	0.59	0.00	1.30	0.01	0.77	1.31	0.01
**Er**	1.72	0.02	1.16	1.68	0.00	3.62	0.05	1.38	3.67	0.04
**Tm**	0.26	0.00	0.00	0.25	0.00	0.53	0.01	1.89	0.53	0.01
**Yb**	1.68	0.02	1.19	1.65	0.00	3.39	0.04	1.18	3.39	0.04
**Lu**	0.26	0.00	0.00	0.25	0.00	0.51	0.00	0.00	0.50	0.01
**Hf**	2.97	0.01	0.34	2.84	0.10	4.92	0.03	0.61	4.97	0.03
**Ta**	0.70	0.04	5.71	0.65	0.00	0.83	0.07	8.43	0.79	0.02
**Pb**	19.97	0.46	2.30	18.88	0.30	10.32	0.35	3.39	10.59	0.17
**Th**	4.92	0.07	1.42	4.80	0.10	5.81	0.12	2.07	5.83	0.05
**U**	2.28	0.04	1.75	2.18	0.10	1.67	0.04	2.40	1.68	0.02

Supplementary Table 1: Trace element averages of certified international reference materials JA-2 and BCR-2 summarised in ref.[80]. Number of runs (n) for each reference material are shown.

	JB-2 (GeoREM), n=3					
	**Mean (ppm)**	**Std.Dev**	**%RSD**	**Ref.**	**Uncert.**	**Accuracy %**
**Li**	8.16	0.16	1.96	8.08	0.15	0.99
**Sc**	55.35	0.52	0.94	54.08	0.76	2.35
**V**	575.27	9.98	1.73	572.40	8.30	0.50
**Cr**	24.63	0.31	1.26	26.65	0.69	7.58
**Co**	37.18	0.58	1.56	37.57	0.67	1.04
**Ni**	13.65	0.29	2.12	14.77	0.51	7.58
**Cu**	222.63	2.76	1.24	222.10	3.60	0.24
**Rb**	6.24	0.25	4.01	6.40	0.11	2.50
**Sr**	175.90	2.21	1.26	178.20	1.50	1.29
**Y**	23.89	0.28	1.17	23.56	0.44	1.40
**Zr**	45.42	0.40	0.88	48.25	0.88	5.87
**Nb**	0.49	0.00	0.00	0.57	0.03	13.27
**Cs**	0.77	0.02	2.60	0.80	0.02	3.75
**Ba**	220.00	2.41	1.10	218.10	2.70	0.87
**La**	2.23	0.02	0.90	2.28	0.04	2.24
**Ce**	6.37	0.05	0.78	6.55	0.09	2.78
**Pr**	1.13	0.01	0.88	1.13	0.02	0.09
**Nd**	6.21	0.01	0.16	6.39	0.06	2.85
**Sm**	2.23	0.01	0.45	2.27	0.02	1.59
**Eu**	0.83	0.01	1.20	0.84	0.01	0.72
**Gd**	3.19	0.03	0.94	3.12	0.05	2.15
**Tb**	0.58	0.01	1.72	0.59	0.01	1.07
**Dy**	3.87	0.03	0.78	3.87	0.06	0.05
**Ho**	0.86	0.00	0.00	0.86	0.02	0.35
**Er**	2.51	0.03	1.20	2.54	0.04	1.06
**Tm**	0.38	0.01	2.63	0.39	0.01	3.31
**Yb**	2.52	0.01	0.40	2.53	0.03	0.36
**Lu**	0.39	0.00	0.00	0.39	0.01	0.15
**Hf**	1.47	0.01	0.68	1.49	0.03	1.14
**Ta**	0.04	0.00	0.00	0.04	0.00	1.01
**Pb**	4.96	0.09	1.81	5.25	0.11	5.52
**Th**	0.26	0.02	7.69	0.26	0.00	0.93
**U**	0.15	0.00	0.00	0.15	0.00	1.83

Supplementary Table 2: Trace element averages of certified international reference material JB-2 summarised in ref.[80]. Number of runs (n) for each reference material are shown.

End Member	PC1	PC2	PC3
**$^{206}Pb/^{204}Pb$**	0.3714	-0.5488	0.4249
**$^{207}Pb/^{204}Pb$**	0.5619	-0.1131	-0.5855
**$^{208}Pb/^{204}Pb$**	0.5727	-0.1835	0.1812
**$^{87}Sr/^{86}Sr$**	-0.3687	-0.5481	0.2860
**$^{143}Nd/^{144}Nd$**	0.2872	0.5933	0.6017
**Variance explained (%)**	53.15	37.42	5.01

Supplementary Table 3: Eigenvectors for the principal components 1-3 when principal component analysis is performed using 5 radiogenic isotope variables. The amount of variance explained by each of the principal components is also included.
